# Role of Silicon in Mediating Salt Tolerance in Plants: A Review

**DOI:** 10.3390/plants8060147

**Published:** 2019-05-31

**Authors:** Yong-Xing Zhu, Hai-Jun Gong, Jun-Liang Yin

**Affiliations:** 1 College of Horticulture and Gardening/College of Agriculture, Yangtze University, Jingzhou 434000, China; yongxingzhu@yangtzeu.edu.cn; 2 College of Horticulture, Northwest A&F University, Yangling 712100, China; gongnavy@163.com; 3 Forewarning and Management of Agricultural and Forestry Pests, Hubei Engineering Technology Center, Yangtze University, Jingzhou 434000, China

**Keywords:** antioxidant enzymes, polyamine, salt stress, silicon, water balance

## Abstract

Salt stress is a major threat for plant growth worldwide. The regulatory mechanisms of silicon in alleviating salt stress have been widely studied using physiological, molecular genetics, and genomic approaches. Recently, progresses have been made in elucidating the alleviative effects of silicon in salt-induced osmotic stress, Na toxicity, and oxidative stress. In this review, we highlight recent development on the impact of silicon application on salt stress responses. Emphasis will be given to the following aspects. (1) Silicon transporters have been experimentally identified in different plant species and their structure feature could be an important molecular basis for silicon permeability. (2) Silicon could mediate salt-induced ion imbalance by (i) regulating Na^+^ uptake, transport, and distribution and (ii) regulating polyamine levels. (3) Si-mediated upregulation of aquaporin gene expression and osmotic adjustment play important roles in alleviating salinity-induced osmotic stress. (4) Silicon application direct/indirectly mitigates oxidative stress via regulating the antioxidant defense and polyamine metabolism. (5) Omics studies reveal that silicon could regulate plants’ response to salt stress by modulating the expression of various genes including transcription factors and hormone-related genes. Finally, research areas that require further investigation to provide a deeper understanding of the role of silicon in plants are highlighted.

## 1. Introduction

Salt stress is one of the main stress factors responsible for declines in crop yield [1]. Currently, more than 20% of the world’s agricultural irrigated land is affected by excess salt concentrations, and this problem continues to worsen worldwide because of improper application of fertilizers, industrial pollution, and poor irrigation practices [2]. Silicon (Si) is the second most abundant element in soil. Although Si has not been classified as an essential element, it has been proven to enhance the quantitative and qualitative traits of plants, especially under environmental stresses, such as salinity, drought, and heavy metal toxicity [3,4]. Moreover, Si can be regarded as “multitalented” element and could ameliorate soil conditions and nutrient contents (e.g., N, P, and K) in plants, making it a high-quality fertilizer for promoting ecologically sound agricultural practices [4,5]. Recently, the progresses and mechanisms of Si in alleviating various biotic and abiotic stresses in plants have been systematically reviewed by several researchers [4,6,7,8,9,10]. However, relatively less attention has been paid to salt stress in these reviews. Rios et al. [11] summarized the improvement effects of Si in plant salinity tolerance mainly from the water uptake and aquaporins points of view. Recently, progresses have been made in elucidating the alleviation effects of Si in salt-induced osmotic stress [12,13] and oxidative stress [14,15]. Moreover, the influence of Si on a putative apoplastic cycling of Na^+^ within the rice root has been further addressed [16]. The direct effect of Si on the salt overly sensitive1 (SOS1) and the high-affinity potassium transporter 1 (HKT1)-mediated Na^+^ efflux has been demonstrated for the first time in maize (*Zea mays* L.) [17]. Therefore, in this paper, we will carry out a review of the major mechanisms by which Si alleviates salinity stress in plants, which provides a more comprehensive update on recent progress and thus provides a theoretical foundation for the practical application of Si-fortified fertilizers in crop production.

## 2. Big Data Analysis of Studies on Effect of Silicon on Stress Alleviation in Plants

**Figure 1:**
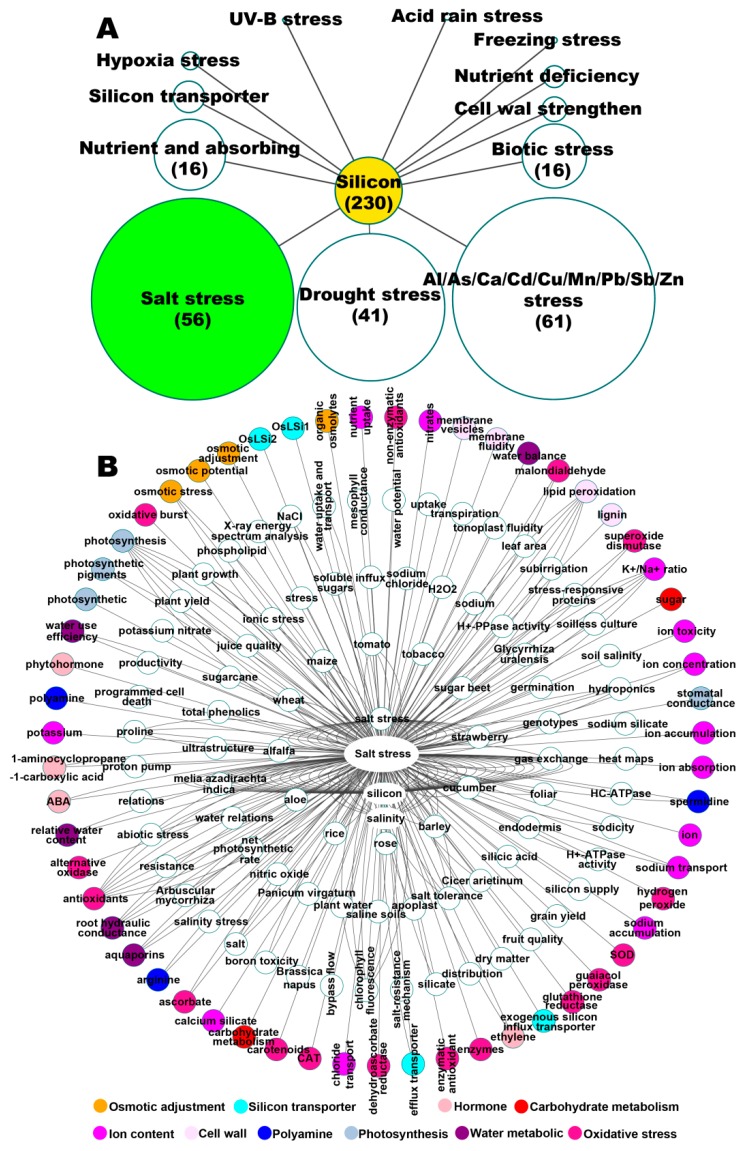
Beneficial effects of silicon (Si) on abiotic stress tolerance and main aspects related to the alleviative effect of Si under salt stress. (**A**) Number of articles related to Si and the alleviative effect of Si on abiotic and biotic stress tolerance published in plant sciences from 1990 to 2018. Articles were mainly collected from Science Direct (https://www.sciencedirect.com/), Spring Link (https://link.springer.com/), PubMed (https://www.ncbi.nlm.nih.gov/pubmed), and Google Scholar (https://scholar.google.com/). (**B**) The network analysis on key words in articles. Articles related to the alleviative effect of Si on salt stress tolerance were selected and the key words of those articles were used to draw this network by Cytoscape (v3.6.0). In the network, those collected key words indicated the aspects (species, substances, physiological processes, alleviation mechanisms, etc.) involved in the alleviative effects of silicon under salt stress. The network was centered with node ‘Salt stress’, which represent ‘articles related to the alleviative effect of Si on salt stress’, and other nodes represent key words in those articles. Number of lines connecting nodes and ‘Salt stress’ represent the number of articles using the words in certain node as key word. As shown in the bottom legend, colored nodes located in the outer ring of the network are grouped into 10 main mechanisms related to the alleviative effect of silicon under salt stress, for example, the blue nodes, including polyamine, spermidine, and arginine, are grouped into polyamine.

To show the current state of research, we collected and summarized the existing studies on the involvement of Si in regulating abiotic stress tolerance using the key words ‘silicon’ and ‘stress’, and found approximately 220 research papers from 1990 to 2019. These studies reported the regulatory effects of Si on plant tolerance to salinity, drought, heavy metal and metal toxicity, cold, hypoxic, nutrient deficiency, ultraviolet radiation, and acid rain stresses; the effects of Si on plant cell wall, nutrition, and absorption; and the cloning of Si transporters and their function in plant stress responses. Our analysis showed that the current research on Si and abiotic stress responses mainly focuses on three aspects: the effects of Si on salinity, heavy/nonheavy metal, and drought stresses. Of these fields, the research on salinity stress is the most systematic and comprehensive, as research materials involved monocotyledons, such as barley (*Hordeum vulgare* L.), wheat (*Triticum aestivum* L.), rice (*Oryza sativa* L.), maize, and sorghum (*Sorghum bicolor* L.); dicotyledons, including cucumber (*Cucumis sativus*), tomato (*Solanum lycopersicum* L.), tobacco (*Nicotiana tabacum*), pumpkin (*Cucurbita maxima*), and peanut (*Arachis hypogaea* L.); and woody plants (e.g., mango (*Mangifera indica* L.) and banana (*Musa spp*.)). The key words of these publications revealed that these studies involved the regulatory effect of Si on water metabolism, photosynthesis, oxidative stress, ion content, hormones, polyamines, and Si transporters (Figure 1). Therefore, in this review, we attempted to describe recent studies related to Si absorption and transport and Si-mediated regulation of ion balance, water balance metabolism, reactive oxygen species (ROS) generation, and photosynthesis, as well as research on the effect of Si using omics-based approaches. We also proposed research directions that urgently require further investigation.

## 3. Silicon Absorption and Transport in Plants

Plants mainly absorb and utilize orthosilicic acid Si[OH]_4_, but Si mainly exists in soil as silica and silicates, most of which cannot be absorbed by plants [2]. The dissolution of Si from soil minerals is a slow process, and the improper use of fertilizer and continuous monoculture of crops has greatly reduced the amount of Si that can be absorbed by plants, resulting in orthosilicic acid deficiency in soils [18]. A more precise understanding of the mechanisms of Si absorption and transport in plants can provide a theoretical basis for the rational use of Si fertilizers.

Even though Si is found in all plants, its content in plants varies among species due to the differences in their Si absorption capabilities [19]. Generally, there are three main mechanisms by which plants absorb Si through root—active, passive, and rejective—and the corresponding types of Si-accumulator plants are as follows. (1) High accumulators, e.g., rice and sorghum. The amount of Si absorbed by these plants is higher than the amount of Si that enters the plant through water uptake. The amount of Si accumulated in the aboveground plant parts as SiO_2_ is greater than 1% on dry weight basis. (2) Intermediate accumulators, e.g., cucumber. The Si absorption rates of plants are equivalent to their water absorption rates. The amount of Si accumulated in aboveground plant parts ranges from 0.5% to 1%. (3) Excluders, e.g., tomato. The Si absorption rates of these plants are lower than their water absorption rates. The amount of Si accumulated in aboveground plant parts is lower than 0.5% [6,20,21]. In addition, the mode of Si accumulation can be classified according to the Si/Ca rations of plants. Plants with a Si/Ca ratio of >1, 0.5–1, and <0.5 are classified as accumulators, intermediates, and excluders, respectively [22]. However, this classification has some inherent problems. An example is that salinity stress affects Ca absorption in plants. Hence, it is not logical to use this standard for the classification of Si-accumulator types [11].

**Figure 2:**
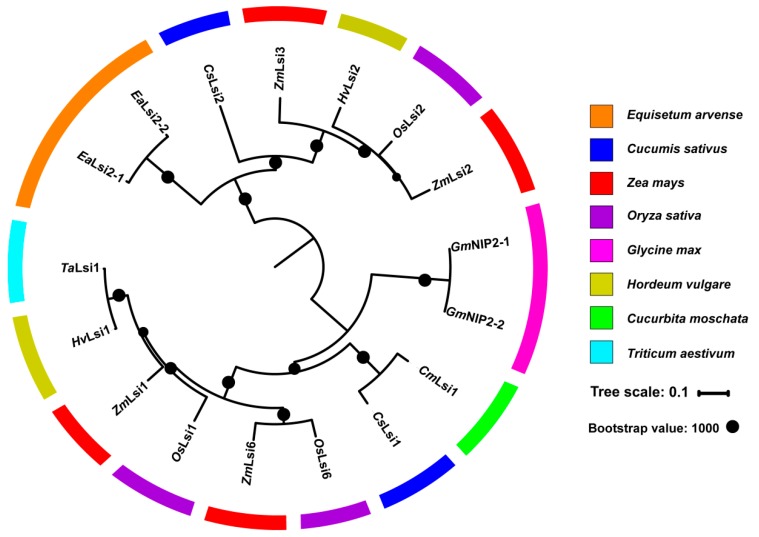
Phylogenetic analysis of plant silicon (Si) transporters identified in *Cucumis sativus* (Cs), *Cucurbita moschata* (Cm), *Equisetum arvense* (Ea), *Glycine max* (Gm), *Hordeum vulgare* (Hv), *Oryza sativa* (Os), *Triticum aestivum* (Ta), *Zea mays* (Zm). Amino acid sequences from 8 members of the Si transporters were aligned using ClustalW2, and a phylogenetic tree was then constructed using MEGA7 program with the maximum likelihood method (Bootstrap value: 1000).

In recent years, some progress has been made on elucidating the mechanisms of Si absorption and transport in plants. Rice Lsi1 (OsNIP2;1) is the first Si transport protein identified in plants. Its homologs belong to the nodulin 26-like intrinsic proteins (NIPs) subfamily of aquaporins (AQPs) [23]. AQPs are small channel-forming transmembrane proteins that belong to the family of membrane intrinsic proteins (MIPs). They are characterized by highly conserved features including the NPA (asparagine–proline–alanine) domain and ar/R (aromatic/arginine) selectivity filter [24]. In rice, the ar/R region of OsLsi1 consists of four small-sized residues, including glycine (G), serine (S), glycine (G), and arginine (R), that form a large constriction pore, allowing relatively large molecules of silicic acid to permeate through [25]. After the first Si transporter being characterized in rice (OsLsi1), more Si transporters (e.g., Lsi2 and Lsi6) have been identified in monocots, such as rice [26], maize [27,28], barley [27,29,30], and wheat [31], and dicots, such as pumpkins [32], cucumber [33,34] and horsetail (*Equisetum arvense*) [35] (Figure 2). 

Localization of Si transporters in root system cells varies among different plant species. For instance, OsLsi1 is localized on the distal side of both endodermis and exodermal layers in the root plasma membrane and is responsible for Si transport from external solution to root cells. On the other hand, OsLsi2 is a putative anion transporter and is located on the proximal side of endodermis and exodermis in the root plasma membrane. This protein is responsible for transporting intracellular Si to the apoplast [23,26]. In barley, HvLsi1 is located in the radicle epidermal and cortical cells, as well as in the plasma membrane of hypodermal cells in the lateral roots. However, HvLsi2 is located only in the plasma membrane of endodermal cells in the root system, and does not exhibit polar distribution [27,29,30]. In pumpkin, a Si influx transporter protein—CmLsi1—was identified and subcellular localization analysis revealed that CmLsi1 is localized on the plasma membrane of all root system cells and does not exhibit polar distribution [32]. Recently, two Si transporter genes—*CsLsi1* and *CsLsi2*—were identified in cucumber [33,34]. The coordination of CsLsi1 (a Si influx transporter) and CsLsi2 (a Si efflux transporter) regulate Si uptake in the roots of cucumber. CsLsi1 is located at the distal side of the endodermis and cortical cells in the root tip and root hairs near the root tips, while CsLsi2 is located on both sides of the root endodermal cells without polarity. Besides Lsi1 and Lsi2, Yamaji et al. [36] and Yamaji and Ma [37] discovered another Si transporter protein in rice, OsLsi6 (NIP2;2), at developmental stages before heading. This protein is mainly expressed on the plasma membrane of the xylem parenchyma cells of the leaf sheath and leaf blades at the side facing toward the xylem vessel. Moreover, OsLsi6 (NIP2;2) is responsible for Si unloading and distribution from the xylem to leaf tissues. At the reproductive stage, *OsLsi6* is highly expressed in node I, the connection between flag leaf and panicles, indicating that Lsi6 is a transporter involved in intervascular transfer of Si in the node of rice plants. In addition, *OsLsi6* is also expressed in the plasma membrane of root tip cells, and its function is not to transport Si absorbed from the root system upward, but to retain Si in the root tips, thereby increasing the tolerance of root systems to water-deficit stress [36,37].

Deshmukh et al. [38] characterized conserved structural features that convey Si influx within the NIPs of 985 AQPs in 25 plant species. They found that a spacing of a specific length—108 amino acids (AA)—between the two NPA domains is an important feature for Si-accumulating plants. For example, 108 AA were found in rice and sorghum, which accumulate more than 3.5% Si in the leaf on the basis of dry weight [39]. Tomato accumulates relatively less Si (only 0.2% Si according to Heine et al. [40]), and has been classified as ‘Si excluder’ alone with a spacing of 109 AA between the two NPA domains [38]. Cucumber is one of the few dicots with a relatively high capacity for Si accumulation (1.4% Si on the basis of shoot dry weight, (see Wu et al. [4]), having 107 AA between the two NPA domains [38]. These results suggested that the distance of amino acids spacing between the NPA domains could be an important molecular basis for the classification of Si accumulators or excluders in plants [38]. However, the field data should also be taken into consideration because the Si transporters that do not belong to the AQPs family might also considerably influence the Si content in the shoot. For example, cucumber Lsi2 (Csa3G182780.1) does not belong to AQPs but functions to transport Si out of the endodermal cells into the stele for xylem loading [34]. Structural feature analysis of Si transporters in more plants will be helpful to decipher the molecular mechanism for Si uptake in plants. Moreover, since the diverse regulatory effects of Si on stress tolerance in plants is partly due to the difference in Si uptake capabilities between different plants, the identification of Si transporters provides numerous alternatives in crop improvement programs, especially for Si excluders like tomato, one of the most consumed vegetables in the world [4,41]. If Si addition could improve seed germination and growth of water-stressed tomato, as suggested in previous studies [4,42], one may expect that genetically increased Si accumulation in tomato could exert better modulation effect on stresses. Until now, more than 400 plant genomes have been released (obtained from NCBI); these genomes could be used to classify plants for Si uptake according to both field data and structural features of Si transporters in more plant species.

Leaves also have the capacity to absorb silicic acid [43]. In several studies, foliar spray of silicate (e.g., K_2_SiO_3_ or Na_2_SiO_3_), silica nanoparticles, and stabilized silicic acid (sSA) have been found to be effective to increase plant growth [43]. For example, Carré Missio et al. [44] reported that foliar application of potassium silicate increased Si deposition on the leaves and reduced coffee leaf rust, which was mainly attributed to the physical role of the polymerized potassium silicate or its osmotic effect against urediniospore germination. Prakash et al. [45] found that foliar silicic acid at 2 and 4 mL·L^−1^ significant increased grain and straw yield. These researches suggested that leaf could absorb silicic acid directly and/or stimulates the plant to absorb more nutrients including Si from the soils, but it still remains obscure about whether transporters involved in this process. More research is needed to understand the mode(s) of absorption and action of Si through foliar application.

## 4. Mechanisms of Silicon in Alleviating Salinity Stress

**Figure 3:**
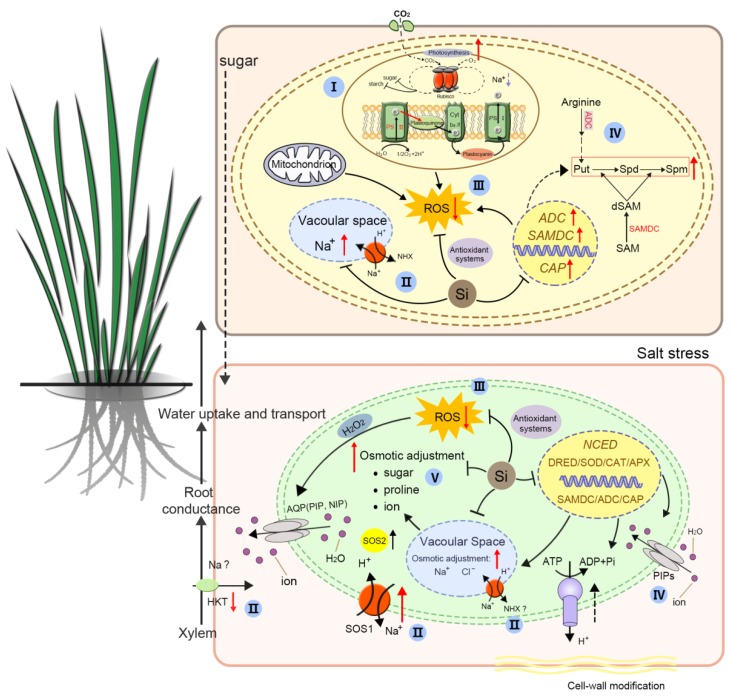
A schematic model for the beneficial impact of silicon on plant under salt stress. Six main strategies are involved in Si’s alleviation of salt stress: For strategy I, Si could enhance photosynthesis by maintaining the integrity of photosynthetic organs, increasing the CO_2_ utilization rate in plants and increasing the openness and activity of the PSII reaction center. For strategy II, Si regulates ion homeostasis through mediating Na^+^ uptake, transport, and compartmentalization, and corresponding gene expression (e.g., *NHX* and *HKT*). For strategy III, Si can regulate the activity/concentration of enzymatic and/or nonenzymatic antioxidants and endogenous polyamine accumulation to alleviate oxidative damage caused by salinity stress. For strategy IV and V, Si enhances the root hydraulic conductance through regulating aquaporin activities and improving osmoregulatory capacities, which contributes to an increase in water uptake and transport. For strategy VI, Si may mediate ion homeostasis and decrease oxidative damage through regulating polyamine metabolism. Single solid black line ended with bar: process of mediating. Single dash black line: speculated mechanisms that need to be experimentally proved. Red arrow: increase (up) or decrease (down). ‘?’ represents mechanisms that are different between species. Chloroplast and mitochondrion component in this schematic model are modified from Yamori [60].

Salinity stress is one of the most common environmental stresses that pose threats to the agriculture industry worldwide. The effects of salinity stress on plants is mainly manifested in the following areas. (1) Osmotic stress caused by excessive soluble salt in the soil decreases the osmotic potential of soil solutions and decreases the ability of plant root systems to absorb water, resulting in physiological drought. (2) Ion toxicity results from the toxic effect of salt ions like Na^+^ and Cl^−^ inside plant cells. Excessive accumulation of intracellular salt ions results in ion imbalance and metabolic disorders. (3) Secondary stresses are caused by osmotic and ionic stresses, including the accumulation of toxic compounds like ROS and disruption of nutrient balances in plants. For example, under high salinity conditions, Na^+^ competes with Ca^2+^ and K^+^ in the cell membrane, resulting in reproductive disorders [46]. In recent years, there have been a large number of reports about the roles of Si in alleviating salinity-induced ion stress and oxidative damage [4,8,9]. Recently, progresses have been made in elucidating the alleviative effects of Si in salt-induced osmotic stress [13], oxidative damage [15], and Na^+^ accumulation [16]. Thus, this review covered the latest research. In addition, on the basis of the available published results, a model describing how Si is involved in alleviating salt stress damage was proposed (Figure 3). We also proposed further studies that are required to address these mechanisms.

### 4.1. Modulation of Seed Germination, Plant Growth, and Photosynthesis

#### 4.1.1. Seed Germination

Seed is a crucial organ in higher plants. Seed germination and early seedling growth signify a key stage in the plant life cycle [47]. Si significantly increases germination characteristics (e.g., germination percentage, germination rate and shoot length) under both normal and stress (e.g., salt/drought) conditions [42,47]. The mechanisms for Si-mediated salt tolerance in seed germination stage still remain obscure, but have been proposed to be associated with the alleviated oxidative stress [42,47]. In mung bean under salt stress, application of Si and salicylic acid (SA) was reported to improve seed physiological quality through increasing K^+^ and decreasing Na^+^ accumulation in seeds [48]. Till now, the interaction between Si and hormones (e.g., abscisic acid and gibberellins), which play a role in controlling seed development and germination, are still not fully understood [49]. Thus, to further reveal the mechanisms of Si-mediated salt tolerance during seed germination, studies are needed to elucidate changes in hormones and hormone-responsive genes in response to salt stress and Si treatment.

The use of Si-uptake mutants can aid researchers to better understand the biochemical function of Si in plant growth and development, in which physical barrier induced by Si deposition on seed surface could be partly excluded. Under salinity stress, Zhang et al. [50] studied the effects of Si on the germination of Si-mutant rice seeds that accumulate less Si in the shoot [51]. Their results showed that the application of exogenous Si increased the bud length, bud weight, and germination rate more obvious in mutant rice than the wild type rice. This might be due to the changes in the mutant seed embryo and seed coat, which enables it to utilize Si more efficiently. The study of Isa et al. [52] found that even though the Si-mutant rice forms relatively less SiO_2_ bodies in the leaves, Si application can still significantly promote the growth of these mutants, suggesting that Si participates in the physiological and biochemical processes of rice. Actually, Laane [43,53] proposed that foliar sSA and nano-SiO_2_ can be classified as biostimulants (‘plant growth promoter’) that enhance nutritional efficacy and decrease abiotic and biotic stresses. The Si-uptake mutants should be investigated further to better understand the alleviating mechanisms of Si, including its potential biostimulant functions, in seed germination under salinity stress.

#### 4.1.2. Plant Growth and Photosynthesis

The enhancement of plant shoot and/or root growth by Si under salt stress have been reported in many plant species, such as rice, maize, wheat, cucumber, tomato, and so on [4]. Roots play a key role in plant development and are the first tissue to perceive salt stress. Si has been reported to regulate root growth and architecture of salt-stressed plants [13,54]. In cucumber, Si was found to increase the root–shoot ratio of salt-stressed plants and improve root hydraulic conductance, likely accounting for improved plant water balance [55]. In rice and sorghum, Si might improve root growth by promoting Casparian band formation and stimulating suberin and lignin biosynthesis or by increasing cell wall extensibility in the growth region [56,57].

Plant growth and yield depend largely on photosynthesis [58]. The salinity stress-induced growth inhibition in plants can be attributed to stress-induced reduction in photosynthesis. From the large amount of data available on the improvement effect of Si on shoot growth and net photosynthetic rate, it is reasonable to speculate that Si might function to maintain a high photosynthetic rate in salt-stressed plants [5,7,59]. The reason why salinity stress results in reduced photosynthetic rate in plants includes the following aspects. (1) Modification of the structure and function of organelles that are responsible for photosynthesis; (2) ion toxicity and oxidative stress to thylakoid membranes and other cellular components; (3) osmotic stress-induced reduction in CO_2_ assimilation rate, which enhances stomatal closure and CO_2_ availability; and (4) inhibition of the transfer of assimilation products [58,60]. Accordingly, the mechanisms by which Si improves plant photosynthesis under salinity stress can be summarized as follows. (1) The addition of Si under salinity stress can decrease ion toxicity and ROS accumulation to maintain the structure and function of organelles that are responsible for photosynthesis [61,62]; (2) The decreased photosynthetic rate is also due to the reduction in stomatal conductance and nonstomatal inhibition, resulting in restricted availability of CO_2_ for carboxylation reactions. Abbas et al. [63] found that Si supplementation in two okra (*Abelmoschus esculentus*) cultivars with different salt tolerance could increase stomatal conductance, transpiration rate, and number and size of stomata, leading to efficient photosynthetic activity under salinity stress. These results showed that Si supplication under salinity stress can improve photosynthesis by maintaining the integrity of photosynthetic organs and photosynthetic pigment levels and by increasing the CO_2_ utilization rate in plants. (3) Last, salinity stress affects the transport and allocation of photosynthetic products. This results in the accumulation of photosynthetic products such as sucrose and starch, causing feedback inhibition of photosynthesis and decreasing plant growth. Currently, relatively few systematic studies have been conducted on the effects of Si on carbohydrate metabolism. In cucumber, Zhu et al. [64] demonstrated that Si application decreased the soluble sugar and starch content in leaves, but increased the starch content in roots through mediating the activities of carbohydrate metabolism enzymes, and thus alleviated photosynthetic feedback repression in leaves and provided more energy storage for root growth. However, experimental evidence is still lacking in this study. Thus, molecular biology approaches should be used to further reveal the mechanisms by which Si affects carbohydrate metabolism.

In recent years, chlorophyll fluorescence parameters have been widely used to study various photosynthetic reactions under stress conditions [65]. Photosystem II (PS II) appears to be a salt stress-sensitive component of the photosynthetic system [66]. In cucumber, salinity stress significantly decreased the F_v_’/F_m_’ (PSII effective photochemical efficiency), F_v_/F_m_ (PSII maximum photochemical efficiency), qP (photochemical quenching coefficient), and Φ_PSII_ (PSII actual photochemical efficiency), whereas it significantly increased the NPQ (non-photochemical quenching coefficient). However, Si application could increase F_v_/F_m_, F_v_’/F_m_’, Φ_PSII_, and qP, and decrease NPQ during salinity stress [13]. Similarly, in salt-stressed aloe, Si application was reported to decrease minimum fluorescence (*F_O_*), and increase variable fluorescence (*F_v_*) and the potential activity of photosystem II (PSII), thus improving photosynthetic efficiency in aloe [67]. These results showed that the addition of Si helps to increase the openness and activity of the PSII reaction center, facilitating the use of more energy in PSII electron transfer and increasing the efficiency of converting light energy into chemical energy [13].

In conclusion, Si enhances photosynthesis in salt-stressed plants by decreasing salt-ion accumulation, scavenging ROS, and regulating carbohydrate metabolism. However, further in-depth research is needed to understand the molecular mechanisms of how Si regulates ROS and carbohydrate metabolism, such as its regulatory effects on the gene expression levels of related enzymes.

### 4.2. Silicon and Ion Homeostasis Regulation

High concentrations of salt ions, particularly Na^+^ and Cl^−^ ions, generally affect the absorption of other nutrients (such as potassium and calcium) by plants and cause increased cell membrane permeability, resulting in metabolism disorders and dysregulation [68]. To cope with salt stress, the plant can reduce Na^+^ uptake, increase Na^+^ efflux, and compartmentalization of Na^+^ in the vacuole to reduce cytoplasmic ion toxicity [69]. Accordingly, the possible mechanisms by which Si regulates ion homeostasis under salt stress can be classified into three main categories as follows.

#### 4.2.1. Silicon Restricts Na^+^ Uptake and Transport

Studies on the mechanisms by which Si alleviates salinity stress in plants are mostly focused on decreasing Na^+^ in the root and/or shoot. For example, the addition of Si to salt-stressed barley could significantly decrease the levels of Na^+^ and Cl^−^ in the root system, with Na^+^ and K^+^ being more evenly distributed throughout the entire root. This has been regarded as one of the major mechanisms by which Si alleviates salinity stress in barley [61]. In salt-stressed alfalfa (*Medicago sativa* L.), Si application significantly decreases Na^+^ levels in the roots, but has no effects on Na^+^ accumulation in the shoot [70]. In wheat, Tuna et al. [71] reported that the addition of Si could simultaneously decrease Na^+^ accumulation in both shoot and root. Garg and Bhandari [72] found that the addition of Si decreases Na^+^ absorption in the root system and translocation toward the leaves, as well as increases the K^+^/Na^+^ ratio, in chickpeas (*Cicer arietinum* L.). However, the mechanisms for these reductions are still largely unknown in most species except in rice. In rice, Si application showed an increased formation of Casparian bands in the exodermis and endodermis [57,73], which might partly hinder the penetration of Na^+^ ions into the symplast and/or transpiration stream [4,74]. Accordingly, Gong et al. [75] showed that in rice (‘IR36’), the addition of Si did not change Na^+^ levels in the root but decreased the upward transport of Na^+^ through apoplastic pathway. However, most recently, Flam-Shepherd et al. [16] measured the radiotracer fluxes of ^24^Na^+^ and proposed that Si does not affect Na^+^ transport across cell membranes and within the bulk root apoplast. Moreover, their study revealed that Si reduced Na^+^ translocation via bypass flow only in the salt-tolerant (‘Pokkali’) rice cultivars, but not in the salt-sensitive (‘IR29’) ones, in which the bypass flow was small and not affected by Si. The decline in the shoot Na^+^ concentration of salt-sensitive (‘IR29’) rice cultivars can be explained by the pronounced stimulation of leaf growth and shoot-to-root ratio. Therefore, much more remains to be explored about the effect of Si on Na^+^ dynamics across membranes and through extracellular space in plants. However, many determinants have not been studied in sufficient detail in salt-stressed plants with or without silicon addition, such as the Na^+^ signal perception process. Moreover, it is unclear whether Na^+^ decrease along with Si addition is due to the changes in the root structure and/or a reduction in the transpiration stream in the xylem, which need to be studied in more species.

#### 4.2.2. The Regulatory Mechanisms Mediating Na^+^ Compartmentalization

Excessive Na exclusion, or its compartmentation into vacuoles, is an important adaptive strategy for plants in response to salt stress [76]. Na^+^/H^+^ antiporter protein plays an extremely important role in Na^+^ efflux and vacuole partitioning [77]. In higher plants, the H^+^-ATPases located on the cell membrane utilize the energy generated from ATP hydrolysis to pump H^+^ out of the cells to generate a transmembrane proton gradient. This provides a driving force for the Na^+^/H^+^ antiporter protein on the plasma membrane. The Na^+^ is expelled out of the cells against an electrochemical gradient when H^+^ is transported into the cells along an electrochemical gradient [78,79,80]. Previous studies showed that compared to the salinity stress alone, Si application significantly increased the activities of H^+^-ATPases on the plasma membrane, as well as the activities of H^+^-ATPase and H^+^-PPase on vacuolar membranes in the roots of barely [79]. The Si-mediated elevation of the H^+^-ATPase activity was conducive for the expulsion of Na^+^ out of the cell, while the elevation in the activities of H^+^-ATPase and H^+^-PPase in vacuolar membranes facilitates the distribution of Na^+^ into vacuoles, thereby decreasing the Na^+^ toxicity in the root [79,80]. However, further studies are required to determine whether Si can directly regulate the activity of the Na^+^/H^+^ antiporter and H^+^-ATPase on the plasma membrane and vacuolar membranes and how. In addition, an excessively high concentration of salt ions in the soil will affect the absorption of other elements (such as nitrogen and calcium) by plants, resulting in ion imbalance, whereas Si has been found to increase the concentration of macroelements, such as Ca, P, and Mg, and microelements, such as B, Fe, Zn, and Mn, in many kinds of plants [2]. Aquaporin has been reported to play important roles in nutrient homeostasis and recent researches suggested that Si could improve plant water content through regulating the activities of root aquaporins, which will be reviewed below.

#### 4.2.3. Potential Interaction between Silicon and Ionic Stress Signaling Pathways

Under salt stress conditions, rapidly sensing excess Na^+^ signal is a prerequisite for initiating the reestablishment of cellular ionic homeostasis [81]. Generally, salt treatment activates the salt overly sensitive (SOS) signaling pathway within a short time period, which is crucial for the regulation of plant ionic homeostasis through extruding Na^+^ into the apoplast [82]. High-affinity K^+^ channel (HKT1) is a key determinant of plant salinity tolerance that may function to mediate Na^+^ influx across the plasma membrane and decrease Na^+^ accumulation in the shoot, thus protecting leaves from Na^+^ toxicity and improving salt tolerance [82]. Until now, little is known about the effect of Si on putatively SOS1-mediated NaCl efflux. More recently, Bosnic et al. [17] proposed that Si decreased Na accumulation in both root apex and cortex of maize. Meanwhile, Si addition allocated more Na^+^ to the leaves via the xylem through upregulating *ZmSOS1* and *ZmSOS2* in the root cortex, but downregulating *ZmHKT1*. Furthermore, Si enhanced sequestration of Na^+^ into the vacuoles by upregulating *ZmNHX*, and thus decreased Na^+^ accumulation in the chloroplasts. This study firstly experimentally demonstrated the direct effect of Si on the expression of SOS and HTK genes. However, the deeper mechanism, such as the detailed regulation mechanisms of Si on SOS signaling pathways, remains obscure. Moreover, it would be interesting to dissect the interaction between Si and other salt stress sensors (e.g., Mitogen-activated protein kinase (MAPK)) involved in salt-induced stress signaling in plants.

#### 4.2.4. Participation of Polyamine in Silicon-Mediated Ion Homeostasis

Recent studies suggested that the effects of Si on the levels of ions, particularly Na^+^ and K^+^, during salinity stress might be associated with the regulation of polyamine metabolism. Polyamines are small aliphatic polycations that are widely distributed in prokaryotic and eukaryotic cells. In higher plants, putrescine, spermidine, and spermine are the most abundant polyamines, and they can be present in different forms, including free, insoluble-bound, and soluble conjugated [15]. During salinity stress, polyamines can regulate the transport of Na^+^ and K^+^ in plants through nonselective ion channels [83]. Recently, the regulatory effects of Si on polyamine metabolism under salt stress have been reported in cucumber and sorghum. In sorghum, Yin et al. [84] found that the addition of Si could increase free and total polyamine levels and decrease Na^+^ accumulation. Moreover, Si application balanced the metabolism of polyamines and ethylene through inhibiting the level of 1-aminocyclopropane-1-1-carboxylic acid (ACC), an important ethylene precursor, to mitigate salt stress. Wang et al. [55] also found that Si had some regulatory effects on polyamine levels (increasing the concentrations of free and conjugated putrescine and free spermidine, but decreasing the concentration of conjugated spermidine), as well as alleviating K^+^/Na^+^ homeostasis in salt-stressed cucumber, suggesting the involvement of polyamine in Si’s reduction of ion toxicity. However, it is still hard to draw the conclusion that Si could mediate the K^+^/Na^+^ homeostasis through regulating polyamines content according to these studies, because under certain conditions, polyamines will not decrease, but promote K^+^ efflux under salinity stress [84]. Therefore, more evidence is required to prove how Si regulates polyamine metabolism and determine under which conditions (e.g., NaCl concentration, stress duration, and seedling age) that Si could regulate polyamine metabolism to balance the K^+^/Na^+^ homeostasis.

Taken together, these studies show that the Si addition can decrease Na^+^ absorption in the root and/or transport to shoot to alleviate the damage caused by salinity stress. However, this mechanism varies between species and varieties and the influence of Si on Na partitioning at the cell, tissue, and organelle levels is still unclear. Moreover, the exact mechanisms of how Si regulates transcription of various mineral transporter genes remain unclear. One may speculate that the regulation of Na^+^ absorption by Si in different species might be affected by the Si absorption ability of roots. In addition, the relationship of SiO_2_–plant cell wall has been well documented in Si accumulators, including monocots and pteridophytes [74]. Coskun et al. [7] proposed an apoplastic obstruction model to explain beneficial effects of Si in plants, which proved a guidance for further study. But the role of Si-induced modifications in the mechanical properties and composition of the cell wall in decreasing Na^+^ uptake and/or transport is largely unknown. On the other hand, some recent studies have shown that interfering with Na^+^ uptake and assimilation is not the only mechanism by which Si alleviates salinity stress damage in plants. For example, in tomato, Romero-Aranda et al. [85] found that addition of Si did not decrease Na^+^ and Cl^−^ concentrations in leaves, but increased the tissue water content to dilute the salt ions that were absorbed. Chen et al. [12] carried out a study in wheat, and found that Si application could alleviate both ion toxicity and osmotic stress caused by salinity stress, with the alleviative effect of the latter being more significant. Zhu et al. [13] reported that the decrease in Na^+^ levels in two cucumber cultivars was not the main mechanism by which Si alleviated salinity stress damage. Apart from ionic stress upon long-term salinity exposure (phase 2), osmotic effect upon short-term salinity exposure (phase 1) reduces the ability of the plant to take up water and inhibit growth rate [82]. However, current studies on Si’s alleviation of osmotic stress caused by salinity stress are limited; this is not conducive to further explore the alleviation mechanisms of Si under salt stress. Therefore, there is an urgent need to examine the regulation of water status in plants by Si under salinity stress, which will be discussed below.

### 4.3. Silicon and Plant Water Balance Under Salinity Stress

The presence of salt in the external soil environment reduces the ability of plants to extract water and this results in physiological drought, which is the major stress affecting plant growth during short-term salinity stress. However, previous studies on Si’s alleviation of salinity stress have mostly focused on the alleviation of ionic stress, and there are relatively few integrated studies on the regulatory effect of Si on water absorption and transport.

#### 4.3.1. Water Relation

Earlier studies showed that Si can decrease the transpiration rate through its deposition on the surface of leaves, thereby decreasing water loss through transpiration and maintaining relative higher water content in plants [86]. However, Si application does not always result in decreased plant transpiration. For example, Gong et al. [75] and Zhu et al. [13] found that Si addition increased the leaf transpiration rate of salt-stressed rice and cucumber seedlings, suggesting a possible role of Si in regulating water uptake in plants. The root system is the first tissue that perceives salt stress and salinity affects the root architecture [81]. Recent studies have revealed that Si addition under salinity stress can increase water content in plants through increasing root water absorption [13,55]. In tomato, Li et al. [87] found that Si promoted root growth and root hydraulic conductance, thereby increasing root water uptake and further improving leaf water content. In sorghum, Liu et al. [88] reported that Si could increase root hydraulic conductance and water absorption by regulating the activity of aquaporins under salt stress. In these studies, an elevation in leaf water status facilitated the maintenance of stomata in an open state, thereby increasing CO_2_ uptake and thus photosynthetic rate. Zhu et al. [13] proposed that Si increased the expression of the main plasma membrane aquaporins in two cucumber cultivars ‘JinYou 1’ and ‘JinChun 5’, thus increasing the root hydraulic conductance under salt stress. This, together with an increased stem hydraulic conductance with Si addition, allows for an increase in leaf water content and finally dilutes the absorbed salt ions. However, it is still unclear whether or not the increase in hydraulic conductance is due to an improvement in the root system structure. Moreover, the effect of silicon on the capacity of the main plasma membrane aquaporins still needs to be confirmed using a molecular biology approach. Wang et al. [55] reported similar results in another cucumber cultivar, ‘JinChun 10’. Nevertheless, Si application not only improved the water content in the leaves of ‘JinChun 10’, it also significantly decreased the Na^+^ content and increased the K^+^ content in leaves, while it was less likely that Si was actively involved in reducing Na^+^ accumulation in ‘JinYou 1’ and ‘JinChun 5’. These differences might be related to different cucumber cultivars (salt stress-tolerant or -sensitive), salinity stress duration, and salt concentrations used in the two studies. Previous studies found that Si deposition on cell walls increases the affinity of xylem vessels for water, thereby affecting water transport capabilities in the xylem [89]. However, Liu et al. [90] pointed out that stem water transport is not the major limiting factor affecting water transport during water-deficit stress in sorghum. Therefore, further studies are required to determine the effects of Si on different transport vessel structures in plants.

In addition, the aquaporin inhibitor, mercury chloride (HgCl_2_) has been used in some studies to prove that Si increases root hydraulic conductance through regulating expression of root AQPs [13,81,90]. For example, in salt-stressed sorghum, Si treatment could significantly increase the transpiration rate, whereas the HgCl_2_ treatment offset the incensement effect of Si in transpiration rate; after recovery induced by dithiothreitol (DTT); however the transpiration rate was higher in Na+Na_2_SiO_3_·9H_2_O-treated seedlings than Na-treated seedlings [90]. However, it is worth noting that Hg^2+^ can act as an inhibitor of both aquaporin and K^+^ channels. K^+^ is an important compatible solute that can regulate water absorption and transport in the root system [91,92]. Besides, not all plant aquaporins are sensitive to Hg, and Hg could have other secondary effects as well [93]. The mechanism by which Si increases root water uptake requires more evidence.

#### 4.3.2. Osmotic Regulation

Osmoregulation is a primary adaptive strategy of plants at the cellular level to derogate the effects of salinity-induced osmotic stress. Salinity stress activates the salt-mediated osmotic stress pathways that induce the synthesis and accumulation of compatible osmolytes (e.g., proline, betaine, and soluble sugars) to increase the osmoregulatory functions of plants [69,94]. Some studies have shown that Si participates in regulating the accumulation of osmoregulatory substances in plants. Examples of this include sorghum and wheat, where researchers found that the addition of Si could significantly alter the soluble sugar and proline content in plants [59,95]. In cucumber, Na+Na_2_SiO_3_·9H_2_O treatment could increase the accumulation of soluble sugars (mainly sucrose and glucose) and decrease the osmotic potential of xylem sap in the root system compared with Na treatment, thus contributing to the promotion of root water uptake [64]. However, this regulatory effect of Si exhibits intercultivar differences, and whether Si participates in enhancing assimilate transport to provide more energy storage in the roots needs to be experimentally proved. Moreover, carbohydrates such as sugars (e.g., glucose, fructose, and fructans) are involved not only in osmoprotection, but also in carbon storage and scavenging of ROS [64].

From the results of these studies, it can be summarized that Si carries out the following functions. (1) Promoting the growth of the root system, (2) regulating aquaporin expression in the root system, (3) improving osmoregulatory capacities, and (4) increasing stem hydraulic conductance. These together facilitate water absorption and transport to aboveground parts, and finally alleviate the osmotic stress caused by salinity stress in plants.

### 4.4. Silicon and Reactive Oxygen Species in Responses to Salt Stress

During salinity stress, one of the immediate responses of plants is overproduction of reactive oxygen species (ROS), such as hydrogen peroxide (H_2_O_2_), superoxide (O_2_^−^), and hydroxyl radicals (OH^.^). The increase of ROS will cause oxidative damage to membranes and organelles [89]. The antioxidant systems include enzymatic and nonenzymatic antioxidants. In plants, enzymatic antioxidants mainly include catalase (CAT), peroxidase (POD), superoxide dismutase (SOD), and ascorbate peroxidase (APX). Nonenzymatic antioxidants mainly include vitamin E, ascorbic acid, and glutathione reductase (GR) [96]. Previous studies have shown that Si could improve ROS scavenging ability by regulating the activities/contents of enzymatic/nonenzymatic antioxidants in plants, and the regulatory effect is different depending on plant species. For example, in barley, Si could increase the activity of CAT, SOD, and GR, but had no effect on the APX activity [62]. In cucumber, the addition of exogenous Si could increase the activities of APX, SOD, GPX, and GR, but had no effects on the CAT activity [97]. In sorghum, Si application has been proposed to reduce the accumulation of H_2_O_2_, which plays a negative role in regulating the activity of aquaporin to enhance aquaporin activity, and thus increase water uptake [88]. Similar results were also found in okra (*Abelmoschus esculentus*) [63], grapes (*Vitis vinifera* L.) [98], wheat [71], tomato [87], and rice [99]. Moreover, the regulatory pattern is different depending upon plant species and Si intensity. For instance, application of Si enhanced AsA-GSH pathway in two rice cultivars differing in salt tolerance, with the ameliorative effect being more pronounced upon Si administration in the sensitive cultivar [100]. One study on *Glycyrrhiza uralensis* showed that the exogenous addition of 1, 2, 4, and 6 mM Si could significantly increase POD activity and reduce malondialdehyde (MDA) concentrations compared to salinity stress alone. However, the SOD activity was only significantly increased when 4 mM Si was used [101]. These results showed that even though the regulatory effects of Si on antioxidant defense systems under salinity stress can vary with plant species, treatment duration, treatment concentration, and growth conditions, overall, Si can decrease the accumulation of ROS through regulating both enzymatic and nonenzymatic antioxidants. However, most of these studies were conducted in the laboratory, which is time- and cost-saving than expensive field trials, but extensive field experiments must be carried out before the application of Si in the field condition and setting any recommendations for farmers because a strong and short time salt stress condition under laboratory conditions could not fully reflect the actual long-term salt stress in field conditions.

Foliar spray of Si, including silicates, stabilized silicic acid, and silica nanoparticles, has been studied in several studies. Similar to root application, foliar sprays with Si compounds are effective in increasing growth and yield and mitigate biotic and abiotic stresses [53]. However, the beneficial effects of foliar silicon on salt stress have been experimentally tested in only a few plants species. In cucumber, foliar spray of SiO_2_ nano fertilizers increased nitrogen (N) and phosphorus (P) content and uptake, and decrease Na content and uptake under salt stress [102]. In salt-stressed *Jatropha integerrima*, foliar application of nano Si increased the growth parameters and decreased the accumulation of Na, Cl, phenolic compounds, and flavonoids in the leaves [103]. Foliar application of Si may be more effective than soil supplement, because silicates supplement in the soil is an indirect source of plant available Si. Moreover, foliar Si feeding could compensate for soil application since foliar spray with Si compounds could promote root growth and nutrition absorption [43]. More research is needed to compare the effectiveness of soil and foliar applied Si.

#### Interactive Effect of Si and Other Substances in Alleviating Salt-Induced Oxidative Damage

Most recently, studies suggested that Si may participate in regulating the antioxidant defense system and relieving oxidative stress through enhanced endogenous polyamine accumulation (mainly spermidine and spermine) [15]. The interactive effects between Si and exogenous substances including mineral element and plant hormone have been reported in several species. In chickpea, Garg and Bhandari [104] evaluated the individual and cumulative effect of Si and arbuscular mycorrhiza (AM) under salinity stress conditions. The results showed that mycorrhiza significant improved Si uptake and Si addition, alone or combined with mycorrhizal inoculation, increased the activities of antioxidant enzymes, such as SOD, CAT, and GPOX, and decreased ROS accumulation under salinity stress. Study of the combination effect of exogenous application of Si and/or other substances like beneficial soil microorganisms and elements in alleviating biotic/abiotic stresses may facilitate the use of Si in more plant species, especially for Si excluders.

Potassium (K) is a macroelement that has been reported to ameliorate adverse effects of salt stress in many species [105]. A great interactive effect between Si and K were reported in improving antioxidant enzyme activity, photosynthetic rate, K uptake, and yield [48]. Selenium (Se), an important micronutrient in animals and humans, functions as a beneficial element in some crops. Studies proved that the combined application of Si and Se was more effective than Si alone in alleviating the toxic effects of salt stress on wheat seedlings through increasing antioxidant enzyme activity and accumulation of osmoprotectants like proline and soluble sugar [88]. Salicylic acid (SA), a plant hormone, is an important signal molecule for modulating plant responses to environmental stresses. Application of salicylic acid and Si has been reported to improve seed quality of mung bean under salinity [48]. Future work is needed to investigate the interactions between silicon and other substance and their coupled response/functionality under salt stress conditions.

Although studies have unraveled the regulatory effect of Si in ROS scavenging, many questions related to its mode of regulation remain unanswered. First and most important, whether this was a primary or a secondary effect of Si on ROS detoxifying proteins (e.g., SOD, APX, CAT, and GPX), and antioxidants such as ascorbic acid and glutathione (GSH) remains unclear from these studies. In the review of Coskun et al. [7], they proposed that there are no biochemical roles for Si(OH)_4_, an uncharged and unreactive molecule, in terms of interactions with enzymes or other intracellular constituents. Therefore, more studies are needed to examine the possible promotion effects of Si on the activities of antioxidant enzymes of plants under stress conditions. Second, metabolic and signaling ROS are shown to accumulate in the different compartments of the cells, mainly chloroplast, mitochondria, peroxisome, and apoplast. Moreover, each set of different biotic and abiotic stress conditions will result in abiotic stress-specific ROS signaling [89,106]. If Si plays an active role in regulating ROS scavenging, it should be further specified when, where, and how (through regulating stress acclimation proteins and enzymes or expressions of genes involved in managing the level of ROS?). Third, it is worth noting that ROS is not always damaging. If cells maintain high enough energy reserves to detoxify ROS, they primarily function as signal transduction molecules that regulate different pathways during plant growth as well as the acclimation of plants to stress [49,96]. How this conflict of ROS production (metabolically or for signaling purposes) and ROS scavenging is resolved in plants is largely unknown, but it is mainly controlled by the ROS gene network [107]. Taking the complex nature of the ROS gene network and its function in plant signal transduction pathways into consideration, the cellular/molecular mechanisms controlling Si-induced/eliminated ROS signaling need to be elucidated.

### 4.5. The Regulatory Effect of Silicon on Genes Expression in Responses to Salt Stress

Owing to the advancement of molecular genetics and genome wide technologies, significant research advances have been made to enhance our understanding of the involvement of Si in increasing stress tolerance. Kim et al. [54] reported that Si application significantly upregulated the expression of genes associated with ABA synthesis (zeaxanthin epoxidase (*ZEP*) and 9-cis-epoxycarotenoid dioxygenase (*NCED1* and *NCED4*)) in rice after 6 and 12 h of the NaCl treatment, but decreased the expression of these genes after 24 h of treatment. In sorghum and cucumber, Si application increased the plasma membrane aquaporin expression in the roots to increase the hydraulic conductance and water uptake ability [81,90]. In tobacco, Liang et al. [108] discovered the cooperation between Si and ethylene signaling pathway. They found that Si application rapidly upregulated the expression of crucial ethylene biosynthesis genes, 1-aminocyclopropane-1-carboxylic acid oxidase (*ACO*) and 1-aminocyclopropane-1-carboxylic acid (ACC) synthase (*ACS*), which increased ethylene levels and regulated plant responses to salinity stress. However, when ethylene was absent, Si did not increase the tolerance of cells to salinity stress, but promoted the production of hydrogen peroxide, leading to cell death. Competition is considered to exist between ethylene and polyamines since they share a common precursor, S-adenosyl-L-methionine (SAM). In sorghum, Yin et al. [84] illustrated that Si modulated the levels (increasing the synthesis and accumulation) of PAs and ethylene by elevating the expression of *ADC* and S-adenosyl-L-methionine decarboxylase (*SAMDC*) genes under salt stress. Both ethylene and PAs are involved in complex signaling systems and play important roles in the regulation of stress tolerance. Exploring the connections between Si and polyamines and other hormones might open up new possibilities to investigate the function of Si at the transcriptional, translational, and molecular levels.

With the development of high-throughput approaches and high-efficiency big data analysis, studies on salt tolerance in plants have extended to genomics, transcriptomics, and proteomics. However, currently there are only a few omics studies available on the Si-induced alleviation of salinity stress. In tomato, Muneer and Jeong [109] found that 40 proteins are differentially expressed under salinity/nonsalinity stress treatments. These differentially expressed proteins are mostly related to stress responses, hormones, transcriptional regulation, etc. In addition, Si could regulate the expression of transcription factor genes (*leDREB-1*, *leDREB-2*, and *leDREB-3*), genes associated with antioxidant enzymes (*leAPX*, *leSOD* and *leCAT* genes), and Si transporter genes (*leLsi-1*, *leLsi-2*, and *leLsi-3*), as well as regulating hormone levels and antioxidative enzyme activities to alleviate the damage caused by salinity stress. In rice, salt stress induced the expression of *Lsi1*, a Si transporter gene. The higher expression of this gene in the tolerant cultivar compared to the sensitive one resulted in the greater Si uptake in the former [110].

High-throughput techniques have been widely used to identify genes or proteins in plants and have been used in several studies to reveal the regulatory roles of Si on gene expressions under non-stresses and stress conditions. For example, under nonstress conditions, Chain et al. [111] and Watanabe et al. [112] found that Si induced the differential expression of only a small number of genes in wheat and rice (47 in wheat and 20 in rice). Similarly, in *Arabidopsis thaliana*, Si application alone induced the differential expression of only two genes [113]. However, some studies have showed that Si upregulates/downregulates a large number of genes under nonstress conditions. For example, Van Bockhaven et al. [114] and Brunings et al. [115] reported that Si application induced the differential expression of 1822 and 221 genes in rice, respectively. This proves that Si plays an important role in normal growth metabolism in rice. The differences between these rice studies might be due to the differences in the sequencing platforms, rice varieties, and culture conditions. Holz et al. [116] employed RNA-seq to study the effects of Si application on gene expression in cucumber tissue culture. Their results showed that Si application induced the upregulation and downregulation of 572 and 564 genes, respectively. A functional analysis showed that these differentially expressed genes mainly participated in photosynthesis, biosynthesis, ion transport, and other primary metabolic processes. Large-scale transcriptome, proteomic, and metabolomic analyses of the effect of Si on salinity stress will help us to gain a system-level understanding of the underlying molecular mechanisms of Si-induced salt tolerance in plants. Most recently, Zhu et al. [117] profiled the transcriptome of cucumber grown in normal condition and salt stress condition with or without Si addition. Si alone caused a large number of genes to be differentially expressed in the leaf of cucumber, including transcription factor, hormone signal transduction pathway-related genes, and genes involved in plant–pathogen interaction, and it may act in a similar manner to an elicitor that preconditioned plants to both biotic and abiotic stresses. Moreover, under stress condition, the expression level of those salt stress-induced differential expressed genes tended to be adjusted back to the control level with Si application. All these studies suggest that omics may be a useful tool to provide an insight into the mechanism for Si-mediated stress tolerance in plants, and further research should be pursued to study the functions of genes that are differentially induced by environment stresses.

## 5. Conclusions and Outlook

The abundance of Si in the Earth’s crust and its beneficial effects on plant growth has cemented its importance in the agriculture industry. Although the alleviative effects of Si on salinity stress have been extensively studied in laboratory and field environments, our knowledge of the molecular and biochemical mechanisms of Si-regulated salt stress response is still very limited. This paper compiles and summarizes these mechanisms into five areas, namely the regulation of ion balance, water status, reactive oxygen species, photosynthesis, and omics studies. Figure 3 summarizes the current knowledge, as described in this review, regarding photosynthesis, ion homeostasis, antioxidants system, polyamine accumulation, and water relationship. However, whether Si regulates these metabolisms directly or indirectly still needs more evidence.

### 5.1. Regulatory Effects of Silicon on Salt Tolerance in Plants Vary Among Species and Cultivars—Why?

Previous studies show that the regulatory effects of Si on salt tolerance in plants vary between species and cultivars. This might be mainly due to the differences in Si uptake capabilities between different plants. Additionally, the Si concentration, stress duration and intensity, cultivation methods used for experimental materials (hydroponics and soil culture), Si application methods (foliar and root application) and forms (silica ions, stabilized silicic acid, and silica nanoparticles), and materials (root, leaf, and stem) used in different experiments also influence the regulatory effects of Si on salt tolerance in plants. For example, stabilized silicic acid (sSA) is the only plant available Si compound, which has been shown to be more effective on plant growth compared with foliar sprays with silicates and has high potentials as an ecofriendly alternative to pesticides [53]. In addition, based on present studies, the alleviative mechanisms of Si on salt-induced ion toxicity and osmotic stress are different. However, in actual operations, it is difficult to distinguish between the two effects. Therefore, in the same species, the response of plants to Si addition can be different at different treatment periods. In spite of this, generally, Si can significantly alleviate the damage caused by salinity stress in many plant species.

### 5.2. Emphasis of Future Study

The identification and characterization of the determinants and regulatory mechanisms of Si in improving plant salt tolerance will help enhance salt stress tolerance in important crop plants. However, many determinants have not been studied in sufficient detail, and some areas require further elucidation. Based on the current published results, we proposed that emphasis of future study could be given to the following aspects. (1) With the development of omics technologies, research on the molecular mechanisms of Si’s alleviation of salinity stress has extended to transcriptome, proteome, and other levels. However, current research is mainly focused on Si‘s alleviation of biotic stress, with only a few preliminary reports available on its effect under abiotic stresses (such as salinity stress). (2) A recent study was the first to experimentally demonstrate the direct effect of Si on the expression of SOS and HTK genes. However, the detailed regulatory mechanisms of Si on SOS signaling pathways and possible interaction between Si and other salt stress sensors remain obscure. (3) More work is needed to analyze the regulatory mechanisms of Si in salt-induced osmotic stress. Moreover, Si transporters belong to the NIP subfamily of the aquaporin family. The cloning and functional studies of Si transporters and AQPs in different species can provide a foundation to better understand the regulatory mechanisms of water metabolism. Furthermore, it is necessary to classify plants (Si accumulator or Si excluder) based on the structural features that convey Si permeability within the NIPs in higher plants. (4) Studies have shown that Si can promote root growth and development of suberized structures in the root endodermis and exodermis and regulate the distribution of ions to different parts of the root under stress conditions. However, the molecular mechanisms by which Si regulates root structure (including cell wall components) and uptake and/or distribution of ions in the root are still unclear and need to be investigated. (5) The accumulation of carbohydrates (such as soluble sugars and starch) can play important roles in energy storage, osmoregulation, and as signaling molecules under salinity stress. Therefore, the regulatory roles and mechanisms of carbohydrates during Si application in stress conditions should be further studied. (6) Foliar-stabilized silicic acid (sSA) can be classified as a biostimulant and has been shown to be very efficient against abiotic and biotic stresses, which deserves much more attention in the future study. In conclusion, we need to expand our basic knowledge of the mechanisms by which Si alleviates salinity stress at the molecular level and establish a theoretical foundation for the practical applications of Si in crop production.
